# Prediction of Muscle Activities from Electrocorticograms in Primary Motor Cortex of Primates

**DOI:** 10.1371/journal.pone.0047992

**Published:** 2012-10-24

**Authors:** Duk Shin, Hidenori Watanabe, Hiroyuki Kambara, Atsushi Nambu, Tadashi Isa, Yukio Nishimura, Yasuharu Koike

**Affiliations:** 1 Precision and Intelligence Laboratory, Tokyo Institute of Technology, Yokohama, Japan; 2 Department of Developmental Physiology, National Institute for Physiological Sciences, National Institutes of Natural Sciences, Okazaki, Japan; 3 Department of System Integrative Physiology, National Institute for Physiological Sciences, National Institutes of Natural Sciences, Okazaki, Japan; 4 Graduate University for Advanced Studies (SOKENDAI), Hayama, Japan; 5 Precursory Research for Embryonic Science and Technology, Japan Science and Technology Agency, Tokyo, Japan; 6 CREST, Japan Science and Technology Agency, Kawaguchi, Japan; University of Montreal, Canada

## Abstract

Electrocorticography (ECoG) has drawn attention as an effective recording approach for brain-machine interfaces (BMI). Previous studies have succeeded in classifying movement intention and predicting hand trajectories from ECoG. Despite such successes, however, there still remains considerable work for the realization of ECoG-based BMIs as neuroprosthetics. We developed a method to predict multiple muscle activities from ECoG measurements. We also verified that ECoG signals are effective for predicting muscle activities in time varying series when performing sequential movements. ECoG signals were band-pass filtered into separate sensorimotor rhythm bands, z-score normalized, and smoothed with a Gaussian filter. We used sparse linear regression to find the best fit between frequency bands of ECoG and electromyographic activity. The best average correlation coefficient and the normalized root-mean-square error were 0.92±0.06 and 0.06±0.10, respectively, in the flexor digitorum profundus finger muscle. The *δ* (1.5∼4Hz) and *γ2* (50∼90Hz) bands contributed significantly more strongly than other frequency bands (*P*<0.001). These results demonstrate the feasibility of predicting muscle activity from ECoG signals in an online fashion.

## Introduction

Brain-machine interfaces (BMI) are versatile technologies with potential to provide assistance to disabled individuals, allowing them greater interaction with the external environment. Several studies have applied electroencephalography (EEG) in the field of non-invasive BMIs: amplitudes of different frequency bands [1], [2]; imagining movement of different parts of the body [3]; slow cortical potentials [4] and gamma band rhythms [5]. Although EEG-based BMIs are generally portable and easy to use in practical application, few studies have tried to reconstruct kinematic information in time series.

Several invasive BMI studies have demonstrated that sequential movements can be reproduced from multichannel spike signals recorded with intracortical multiple electrode arrays [6]–[13]. They have reported that multichannel spike signals are effective in predicting kinematic information such as direction and velocity of the arm. However, although intracortical electrodes can provide rich information for the control of BMIs, they face limitations such as signal degradation due to glial scarring [14] and potential displacement from the recording site [15].

Electrocorticography (ECoG) is an alternative approach to less invasive BMIs [15]–[23]. Since ECoG records directly from neuronal activities on the cortical surface, ECoG has higher spatio-temporal resolution with better signal-to-noise ratio than scalp EEG [24], [25]. ECoG has also shown potential as a stable long-term recording method [21]. Several studies using ECoG have already succeeded in the classification of movement direction [16], [17], grasp type [22], and prediction of hand trajectory [18], [20], [21].

Despite these successes, however, there still remains considerable work for the realization of ECoG-based prosthetics. The human neuromuscular system naturally modulates mechanical stiffness and viscosity to achieve proper interaction with the environment. Current rehabilitation robots can perform sophisticated operations including stiffness control [26], [27]. Our previous works suggested that the angle, torque, and stiffness of joints can be predicted from muscle activity [28], [29], [30]. Therefore, decoding muscle activity is an important component for realizing BMI systems capable of controlling interaction force or stiffness.

The aim of this study is to predict time-varying muscle activities from ECoG signals as a basis for a neuromuscular BMI system. Two well-trained Japanese monkeys performed a series of reaching, grasping, pulling, and releasing movements. We simultaneously recorded 15 or 16 ECoG signals of the primary motor cortex (Ml) and 12 electromyography (EMG) signals in the right arm. We predicted EMG from ECoG signals using sparse linear regression (SLiR). We also examined the weights of the prediction model in order to infer which sensorimotor rhythms contribute more to the prediction. Our results indicate that multiple muscle activities can be accurately predicted from a small number of ECoG signals.

## Methods

### Ethics statement

All experimental procedures were performed in accordance with the Guidelines for Proper Conduct of Animal Experiments of the Science Council of Japan and approved by the Committee for Animal Experiment at the National Institutes of Natural Sciences (Approval No.: 11A157). Monkeys were monitored closely and animal welfare was assessed on a daily basis and, if necessary, several times a day. This included veterinary examinations to ensure animals were not suffering as well as the use of analgesics, antiemetics, or antibiotic therapy if necessary. No monkeys were sacrificed in this study. Animals were housed individually on a 12-hour light/dark cycle and provided a rubber toy. They were not food deprived. Water was provided in their home cage and recording booth. The animal welfare and steps taken to ameliorate suffering were in accordance with the recommendations of the Weatherall report [31], “The use of non-human primates in research.”

### Behavioral task

**Figure 1:**
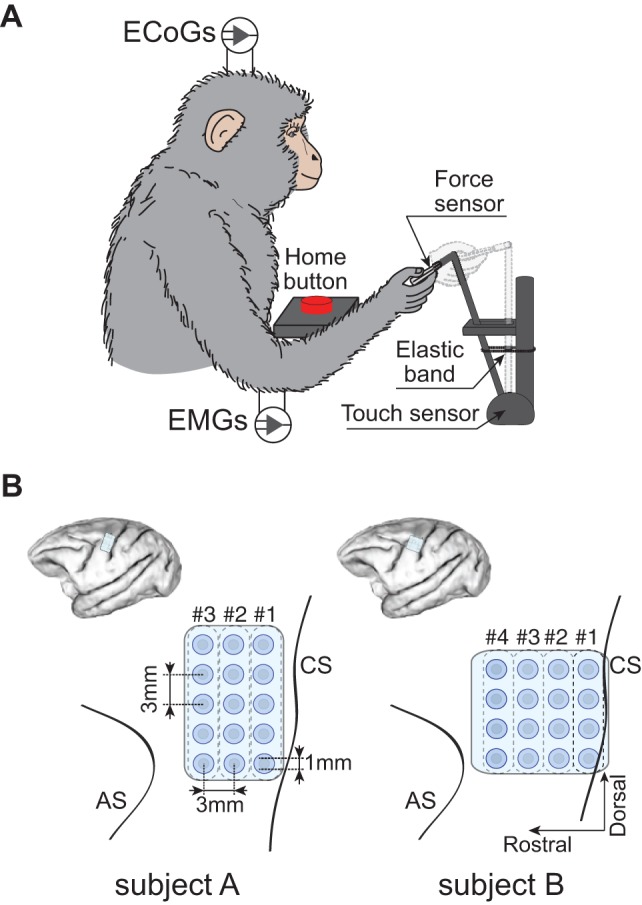
Behavioral task and ECoG electrode locations. A) Monkeys performed sequential right arm and hand movements, which consisted of reaching to a knob, grasping the knob with a lateral grip, pulling the knob closer, releasing the knob, and returning the hand to the home position, in a 3-D workspace. During the task, ECoG and EMG signals were recorded simultaneously. B) Schematic diagrams of ECoG electrode locations in left hemisphere. The planar-surface platinum electrode arrays were placed on the gyrus between the central sulcus (CS) and the arcuate sulcus (AS) in the primary motor area. The # indicates the location according to the column of ECoG electrodes.

**Figure 2:**
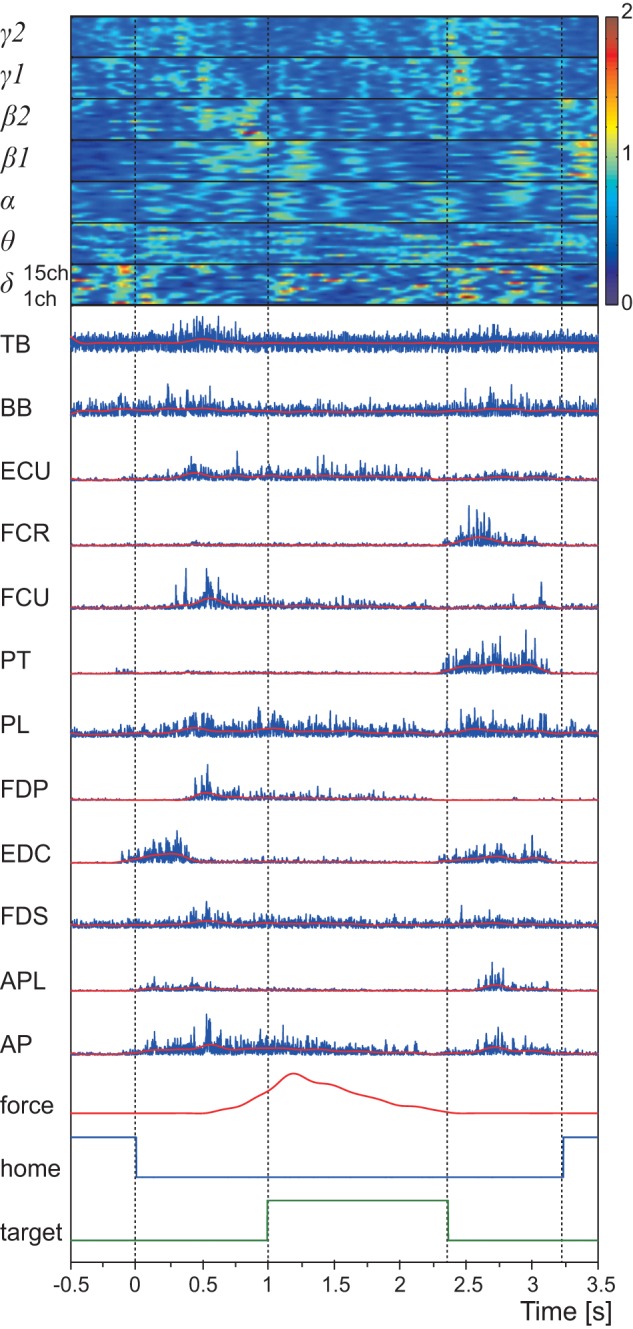
Example of measured signals from monkey A during the tasks. All signals are aligned to trial onset. At the top are frequency feature values of the ECoG signals. Frequency features were resorted in time and sensorimotor rhythm bands. Frequency features of each band are ordered by channel. Below the frequency features are the 12 EMG signals recorded from wire electrodes implanted into muscles of the right forelimb. The blue traces represent original muscle activities. The red traces represent muscle activities obtained by low pass filtering (cut-off frequency: 4 Hz). Below the EMG are grip force on the knob and logical signals, indicating presence of the monkey's hand on the home button or grasping the knob.

Two Japanese macaques (Monkey A: male, at 8.9 kg; Monkey B: female, at 4.7 kg) were trained to perform reaching and grasping tasks with the right hand as shown in Figure 1A. The grasping object was a small plastic knob instrumented with a thin-film force sensor (FlexiForce; Tekscan, Inc., South Boston, MA) to measure grip force. The knob was attached to the end of a joystick switch lever equipped with several elastic bands. The joystick switch detected the pulling duration (positive phase of the target signal in Figure 2). The monkey launched a trial by placing its hand on a home button located in front of the chair for a predetermined length of time. If the monkey held the home button for 2 s, a “go” cue was given in the form of a beep sound instructing the monkey to reach for the knob. The monkey then had to pull the knob and hold for a preprogrammed length of time. The monkey would then release the knob and return its hand to the home button. When the monkey successfully pushed the home button to elicit a go cue and pulled the knob to the required displacement of 6 cm, it received a juice reward. The monkeys performed this task repeatedly, with monkey A performing a total of 134 trials and monkey B performing a total of 248 trials. Monkey B performed one additional session that was used to test the proposed method on continuous rather than trial-based data.

### Surgery for ECoG and EMG electrode implantation

Both monkeys underwent surgery on different days to implant an ECoG electrode array and EMG wire electrodes under anesthesia after they completed behavioral training. The monkeys were anesthetized with ketamine (1.0 mg/kg) and xylazine (0.5 mg/kg). The inhalation of 1–2% isoflurane maintained anesthesia during the surgeries. We also continuously monitored electrocardiogram, pCO_2_, and arterial O_2_ levels.

We chronically implanted a platinum ECoG array (Unique Medical Corporation, Tokyo, Japan) over the left primary motor cortex (M1), which had 15 (monkey A: 5×3 grid) and 16 (monkey B: 4×4 grid) channel electrodes, as shown in Fig 1B. The electrodes had a diameter of 1 mm and an inter-electrode distance of 3 mm center-to-center. Four silver wires (300 μm in diameter; over 5 cm in length) were used as reference and ground electrodes and shunted (single-end mode) through connectors (#A8828-001; Omnetics Connector Corporation, Minneapolis, MN, US). A craniotomy was performed above M1, and the dura was incised and reflected. We placed the ECoG array on the rostral bank of the central sulcus, the hand/arm area of M1. The dura was closed using 6.0 synthetic absorbable suture threads within surgical glue composed of gelatin after the two silver wire reference electrodes were inserted into the subdural space. A piece of artificial dura mater was applied over the dura and two reference ground electrodes were inserted into supradural space between the dura and the skull. The craniotomy was closed with a piece of dental acrylic, and head holders were attached to the skull. Finally, the skull was coated with dental acrylic.

EMG activities of the right forelimb muscles were recorded from chronically implanted pairs of multi-stranded stainless steel wires (Cooner Wire, Chatsworth, CA, USA). They were subcutaneously tunneled to the following target muscles: adductor pollicis (AP), abductor pollicis longus (APL), flexor digitorum superficialis (FDS; monkey A only), flexor digitorum profundus (FDP), and extensor digitorum communis (EDC) for hand muscles; flexor carpi radialis (FCR; monkey A only), flexor carpi ulnaris (FCU), palmaris longus (PL; monkey A only), extensor carpi radialis (ECR; monkey B only), and extensor carpi ulnaris (ECU; monkey A only) for wrist muscles; and pronator teres (PT; monkey A only), biceps brachii (BB), and triceps brachii (TB) for elbow muscles. Isolation of the muscles was confirmed with electronic current stimulation through a silver ball electrode (intensity of a single monophasic current pulse: ∼100 μA, pulse duration: 1ms, 1 stimulation: 5 pulses with 3ms pulse intervals, inter-stimulus interval: 1s), which evoked joint movement and muscle twitch in the fingers and arm during electrode implantation surgery. In each muscle, two electrodes were implanted and one electrode was used as reference (differential mode). Circular connectors (MCP-12, Omnetics, Minneapolis, MN, USA) were anchored to the skull.

### Data recording

Recording sessions were initiated two months after the surgery. ECoG and EMG signals were sampled at 4 kHz using an acquisition processor system (Plexon MAP system; Plexon, Inc., Dallas, US). ECoG signals were filtered with band-pass filters through multi-channel bio-signal amplifiers (monkey A: 1.5 Hz high-pass and 1 kHz low-pass analog filters, MEG-6116, Nihon Kohden Corporation, Tokyo, Japan; monkey B: 0.7 Hz high-pass and 8 kHz low-pass analog filters, Plexon, Inc., Dallas, USA). Due to logistical reasons, two different amplifiers were used for ECoG filtering in the two subjects. However, post-hoc data processing (Results section) showed no substantial differences between the band-pass filters of the two amplifiers. EMG signals were also filtered online with 1.5 Hz high-pass and 3 kHz low-pass analog filters through a signal amplifier (MEG-6116: Nihon Kohden Corporation, Tokyo, Japan). Separate amplifiers were used for signal filtering because the analog-to-digital converter boards of the acquisition processor system did not support user-defined filters.

### Preprocessing of ECoG and EMG data

ECoG signals were down-sampled to 500 samples per second to match movement data and re-referenced using a common average reference (CAR) montage. Bidirectional fourth-order Butterworth band-pass filters were applied to each ECoG signal, dividing them into specific rhythmic bands. These bands were *δ* (1.5∼4 Hz), *θ* (4∼8 Hz), *α* (8∼14 Hz), *β1* (14∼20 Hz), *β2* (20∼30 Hz), *γ1* (30∼50 Hz), and *γ2* (50∼90 Hz). We selected these particular frequency bands, because their use is common in current EEG and ECoG based BMIs. The seven band-pass filters split each of the 15- or 16-channel ECoG signals into seven band-passed signals to produce *M* channels of band-pass filtered signals. Each bandpass filtered signal 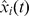 was then normalized by the standard z-score, resulting in signal sources 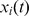, where
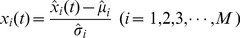
(1)and 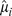 and 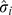 are the mean and the standard deviation of 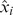, respectively, over a 1 s interval before the time *t*. Finally, the resulting amplitude modulations were smoothed with a Gaussian filter (width: 0.1 s, σ: 0.04 s).

EMG signals were rectified and passed through a 4th-order low-pass filter with a cut-off frequency of 4 Hz and further down-sampled to 500 Hz, resulting in muscle activities.

### Prediction of muscle activities from ECoG signals

Previous studies have reconstructed finger-movements, finger force, and arm EMG patterns from neural firings [32], blood oxygen level-dependent signals [33], near-infrared spectroscopy signals [34], cortical current dipoles [35], EEG signals [36], and local field potential (LFP) signals [37]. Since the SLiR algorithm can automatically select significant input variables and reduce the number of input dimensions, we used the Variational Bayesian Sparse Regression toolbox [38] to determine which band is effective in predicting EMG signals. The SLiR algorithm estimates the linear weight and automatic relevance determination (ARD) parameters [39], which represent how the weight contributes to the reconstruction. Based on these ARD parameters, SLiR identified only the channels that provided the best generalization properties by pruning the channels ineffective for reconstruction (setting the linear weight value to zero) [40].

The predicted muscle activity at time *t*, is described as 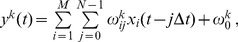
(2)where 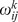 is the weight coefficient of the *k*-th muscle for the *i*-th signal source at a delay time 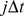, 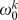 is the bias term, 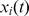 is the *i*-th ECoG source at time *t*, and 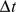 is a discrete-time step-size of 20 ms. The muscle activity at time *t* was predicted using 10 time points (*N* = 10) starting 200 ms before the target time *t*.

### Analysis

Accuracy of the muscle activity prediction was evaluated using 10-fold cross validation. We extracted trials in reference to the trial onset, where onset was defined as the movement initiation from the home button. The extracted trials were randomly partitioned into 10 subsets. Each subset for monkeys A and B had 12 trials and 24 trials, respectively. A single subset was retained as a test subset to evaluate the model, and the remaining 4 subsets were used as training data. The cross-validation process was then repeated 10 times, with each of the 10 subsets used exactly once as a test subset. To verify its applicability to continuous data, we also tested the model on 50 s of task data randomly extracted from an additional session performed by monkey B.

We calculated the coefficient of correlation (CC) to evaluate the similarity between actual and predicted muscle activities. Accuracy was also evaluated using normalized root-mean-square error (*nRMSE*) between actual and predicted muscle activities, defined as
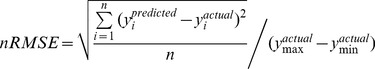
(3)where for each time *i* (*i = *1, 2, *… ,n*), 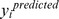 is the predicted muscle activity and 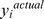 is the actual muscle activity, and 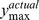 and 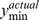 are the maximum and minimum of actual muscle activities, respectively.

### Weight values for contributing to prediction

We examined the weight values for frequency bands, locations, muscles, and subsets in the prediction model. Weight coefficients 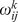 were averaged across time points (j = 0, 1, 2, ..., 9). They were normalized by the maximum weight within each muscle and applied in a 3-way ANOVA. We used locations rather than electrodes because the 3-way ANOVA could not be calculated with so many degrees of freedom. Since Rathelot and Strick [41] reported that corticomotoneuronal (CM) cells that make monosynaptic connections to spinal motoneurons are located predominantly in the anterior bank of the central sulcus, we hypothesized that the weight values of locations near the central sulcus would be larger than those of the other locations. Median differences were then analyzed using Tukey multiple comparison tests. Statistical significance was assessed at a 5% or 1% confidence level using an F test. F_φ, ρ_ values (Results section) represent the ratio of variances within subjects with degrees of freedom φ and between subjects with degrees of freedom ρ. Large F values indicated more variance between subjects than within subjects. All data processing and analyses were performed using MATLAB R2011b (The Mathworks, Inc., Natick, MA, USA).

## Results

### Multiple task-related muscle activities

Movement duration averages and standard deviations (STD) for monkeys A and B were 3.22±0.24 s and 1.16±0.29 s, respectively. Therefore, we set the duration of each trial to 4.0 s (monkey A) and 2.0 s (monkey B), including a before-onset period of 0.5 s and after-onset periods of 3.5 s (monkey A) and 1.5 s (monkey B). Figure 2 shows an example trial including frequency band features of the ECoG signals, rectified raw EMG signals, grip force, and logical signals. Sequential movements were divided into four movements according to the logical signals. Four patterns of actual muscle activity were observed when subjects performed the sequential movements. First, activity for the elbow flexor muscle BB and finger extensor EDC increased before trial onset to raise the hand. Second, all muscles except the wrist flexor muscle FCR and the wrist pronation muscle PT peaked upon opening the hand to grasping the knob. Third, the palm muscle PL, finger flexor muscles FDP and FDS, and thumb adductor muscle AP were activated while the monkey grasped the knob (from 1 s to 2.45 s). The wrist muscle ECU and FCU also co-contracted to fix the wrist during the knob pulling movement. Fourth, all muscles, with the exception of FCU, FDS and FDP, peaked when the monkey released the knob and returned its hand to the start button. Muscle activations almost always occurred in this pattern, though timings slightly differed from trial to trial.

### Reconstruction using sparse linear regression

**Figure 3:**
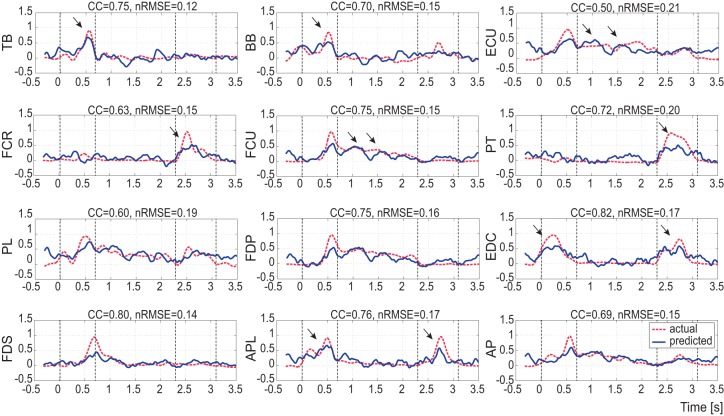
Representative example of predicted and recorded muscle activities. Dotted lines are actual muscle activities from EMG signals measured with wire electrodes, and solid lines represent predicted muscle activities from ECoG signals of monkey A. The normalized root mean square error (*nRMSE*) and correlation coefficient (CC) are also shown above each panel.

Figure 3 shows typical plots of predicted muscle activity (solid line) from a test subset in comparison with actual muscle activity (dotted line) during a trial conducted with monkey A. The proposed method was able to predict sequential muscle activations during the reaching and grasping task, as well as concurrent bursts such as the EDC and APL. In particular, the proposed method generated the co-contraction features occurring for grasping, as seen in the ECU and FCU.

CC and *nRMSE* between the actual and predicted EMGs were used to quantify the information extracted directly from ECoGs related to muscle activity. The highest CC values were 0.73±0.10 for EDC in the 10^th^ test subset for monkey A and 0.92±0.06 for FDP in the 3^rd^ test subset for monkey B. The highest *nRMSE* values were 0.16±0.02 for FDS in the 3^rd^ test subset and 0.06±0.10 for FDP in the 8^th^ test subset for monkeys A and B, respectively. Table 1 summarizes the results of the validation in predicting each muscle for 10 test subsets of monkey B (see also Table S1 for monkey A). Grand averages and standard error of the mean (SEM) for each muscle ranged between 0.55±0.013 and 0.88±0.009 for CC and 0.17±0.003 and 0.42±0.007 for *nRMSE*. These results clearly show that ECoG data contained information about muscle activations.

**Table 1:** Summary of prediction accuracies for 10-fold cross validation of monkey B.

Statistics	Test set	TB	BB	ECR	FCU	EDC	FDP	APL	AP
CC	1	0.51±0.40	0.86±0.12	0.59**±**0.32	0.81±0.21	0.81**±**0.12	0.89±0.17	0.86±0.12	0.84±0.17
	2	**0.62±0.36**	**0.87±0.11**	0.57±0.34	0.83±0.14	0.80±0.14	0.86±0.20	0.85±0.17	0.85±0.11
	3	0.56±0.33	0.83±0.25	0.58±0.30	0.83±0.14	0.83±0.15	**0.92±0.06**	0.86±0.17	0.87±0.13
	4	0.51±0.38	0.85±0.25	0.54±0.32	0.77±0.25	0.83±0.12	0.85±0.24	0.85±0.15	0.85±0.22
	5	0.54±0.35	0.84±0.29	0.56±0.33	0.80±0.21	0.80±0.14	0.86±0.23	0.83**±**0.19	0.80±0.23
	6	0.51**±**0.39	0.87**±**0.11	**0.60±0.32**	0.82±0.20	**0.83±0.10**	0.89±0.17	**0.87±0.11**	0.85**±**0.17
	7	0.61±0.36	0.86±0.12	0.55±0.34	0.83±0.15	0.79±0.15	0.86**±**0.19	0.83±0.18	0.85±0.12
	8	0.57±0.33	0.83±0.25	0.59±0.30	**0.84±0.14**	0.82±0.15	0.92±0.06	0.86±0.18	**0.88±0.12**
	9	0.54±0.38	0.85±0.25	0.56±0.32	0.78±0.24	0.83±0.12	0.86±0.23	0.86±0.15	0.86±0.21
	10	0.57±0.38	0.87±0.11	0.56±0.26	0.79±0.21	0.80±0.12	0.85±0.25	0.84±0.18	0.86±0.13
	Avg.	0.55±0.013	0.85±0.006	0.57±0.007	0.81±0.008	0.82±0.006	**0.88±**0.009	0.85±0.004	0.85±0.007
***nRMSE***	1	0.40±0.31	0.12±0.13	0.32±0.11	0.21±0.20	0.12±0.05	0.17±0.06	0.12±0.08	0.17±0.09
	2	0.36±0.27	**0.11±0.07**	0.34±0.11	**0.14±0.41**	0.14±0.06	0.20±0.25	0.17±0.08	**0.11±0.12**
	3	0.33±0.30	0.25±0.21	0.30±0.12	0.14±0.13	0.15±0.05	0.06±0.10	0.17±0.10	0.13±0.06
	4	0.38±0.23	0.25±0.43	0.32±0.11	0.25±0.16	0.12±0.08	0.24±0.23	0.15±0.09	0.22±0.16
	5	0.35±0.18	0.29±0.44	0.33**±**0.09	0.21±0.21	0.14±0.06	0.23±0.19	0.19±0.11	0.23±0.17
	6	0.39±0.30	0.11±0.13	0.32±0.11	0.20±0.20	**0.10±0.05**	0.17±0.065	**0.11±0.08**	0.17±0.09
	7	0.36±0.29	0.12±0.07	0.34±0.11	0.15±0.39	0.15±0.07	0.19±0.24	0.18±0.09	0.12**±**0.12
	8	**0.33±0.30**	0.25±0.22	0.30±0.12	0.14±0.13	0.15±0.05	**0.06±0.10**	0.18±0.10	0.12±0.06
	9	0.38**±**0.23	0.25**±**0.42	0.32±0.11	0.24**±**0.17	0.12**±**0.08	0.23±0.23	0.15±0.09	0.21±0.16
	10	0.38±0.38	0.11±0.08	**0.26±0.11**	0.21±0.10	0.12±0.07	0.25**±**0.07	0.18±0.12	0.13±0.10
	Avg.	0.42±0.007	0.21±0.010	0.31±0.004	0.28±0.010	0.19±0.004	0.17±0.008	**0.17±0.003**	0.20±0.008

Each cell except Avg. shows the CC or the ***nRMSE*** (mean ± STD) of 24 trials. Bold numbers indicate the best value in each test subset. The Avg. cells show the grand averages of mean and SEM. Bold numbers here indicate the best grand averages.

A one-way ANOVA was conducted to judge whether performance of the proposed method differed significantly among the test subsets. Significant differences of the *nRMSE* values were not observed among test subsets in both monkeys (monkey A: *F*_9, 110_ = 0.65, *p* = 0.75; monkey B: *F*_9, 110_ = 0.03, *p* = 1).

**Figure 4:**
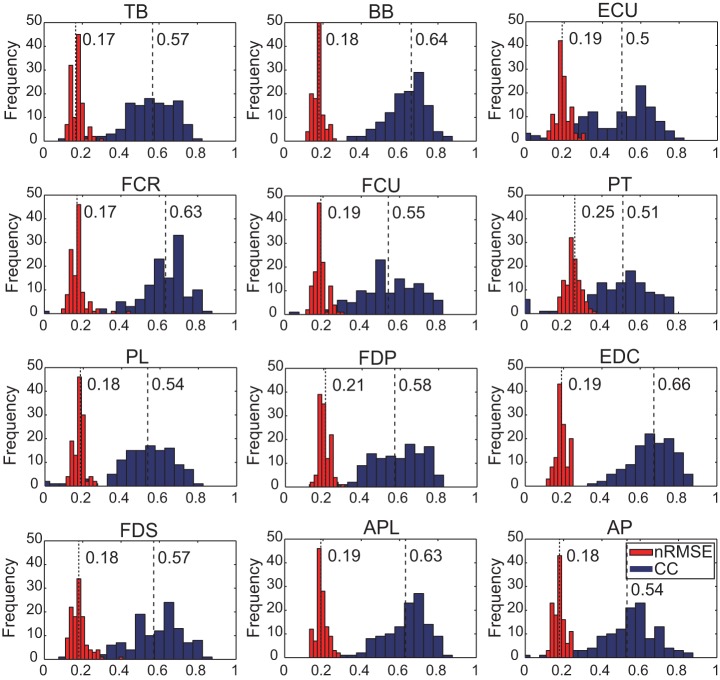
CC and *nRMSE* distributions for each muscle of monkey A. The height of each blue bar is equal to the CC density of the interval (0.05). The height of each red bar is equal to the nRMSE density of the interval (0.02). The total area of the histogram is equal to the number of trials used as validation data. Each dotted line with a number shows the median of *nRMSE* or CC for each muscle. For visualization, we substituted zeros for all negative CC values in validation.

The histogram in Figure 4 shows the distribution of CC and *nRMSE* for each muscle of monkey A. Average and STD of the median of CC were 0.59±0.04 and 0.85±0.07 for monkeys A and B, respectively. Average and STD of the median of *nRMSE* were 0.19±0.01 and 0.18±0.03 for monkeys A and B, respectively. Negative CC values were not removed from the analysis but were substituted with zeros strictly for visualization in Figure 4.

**Figure 5:**
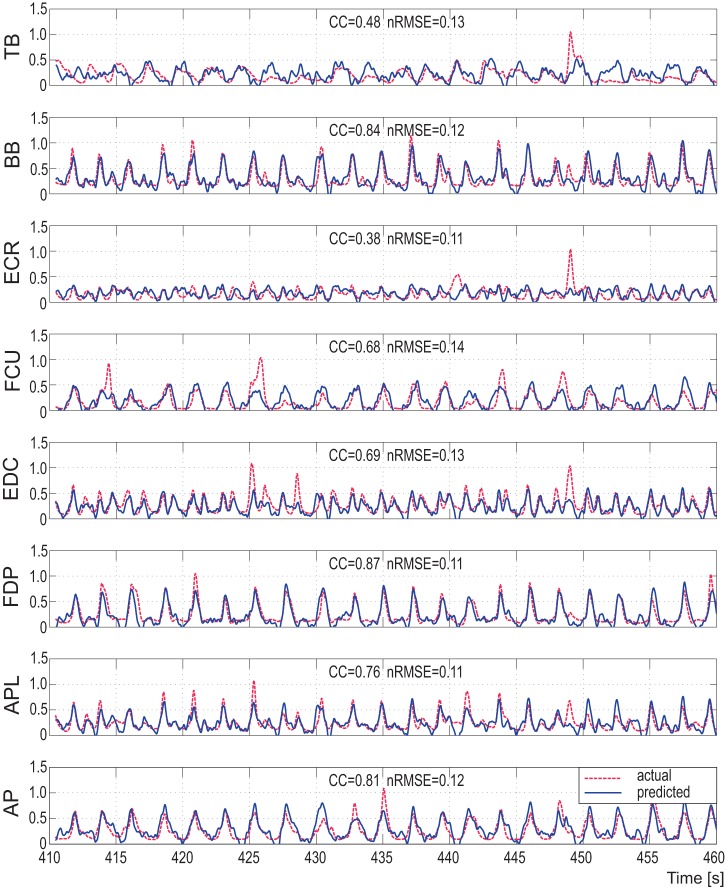
Example of muscle activity prediction in a continuous time series from monkey B. Dotted lines are actual muscle activities from EMG signals and solid lines are predicted muscle activities from ECoG signals over a 50 s time interval. Both lines were normalized to the ranges of actual muscle activities. The normalized root mean square error (*nRMSE*) and correlation coefficient (CC) are also shown.

We also applied the prediction model to continuous data from an additional session by monkey B. One example of continuous prediction is shown in Figure 5, where the prediction was stable even for repetitive trials over 50 s. Mean and STD of CC and *nRMSE* for each muscle ranged from 0.38±0.08 to 0.87±0.02 (CC) and 0.11±0.01 to 0.17±0.05 (*nRMSE*). These results clearly show that the model can predict muscle activity from ECoG in an online fashion.

### Frequency bands contributing to prediction

**Figure 6:**
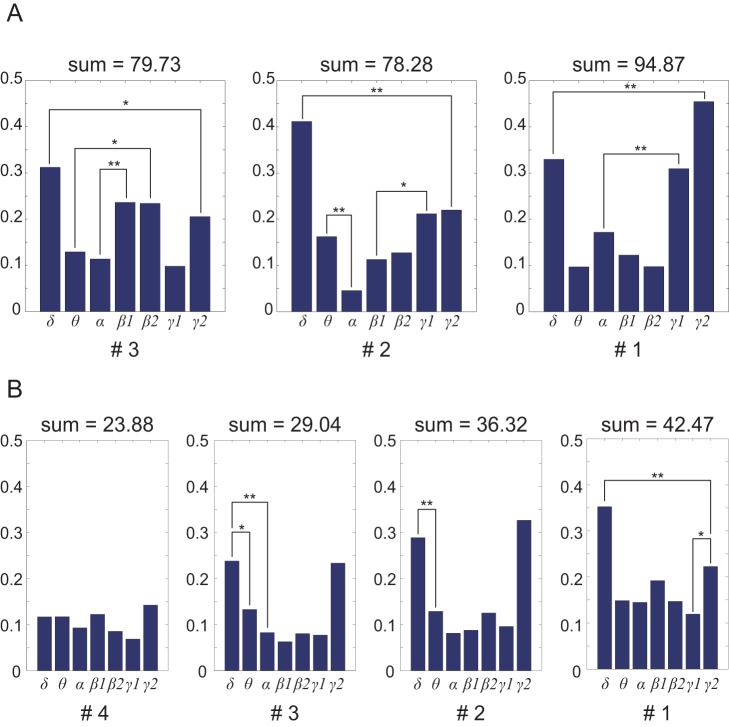
Contribution of each frequency band for EMG prediction. Each panel shows results of multiple comparisons among the frequency bands for each location level (A: monkey A, B: monkey B). Each bar represents the mean of the median weights of each frequency band. At the top of each graph are the sum of the total weights. The # indicates the location of the ECoG electrodes. Noteworthy significant differences between weight values of frequency bands are marked with * (*p*<0.05) and ** (*p*<0.001). Other significance comparisons are omitted for visualization purposes.

We analyzed the weight values for the 7 frequency bands, 3 or 4 locations, 12 or 8 muscles, and 10 subsets in the prediction model. A 3-way ANOVA was conducted to test the effects of three factors (frequency bands, locations, and muscles). Results of ANOVA showed significant main effects of frequency bands (*F*_6, 1008_ = 43.46, *p* = 2.42×10^−47^; *F*_6, 672_ = 33.78, *p* = 1.07×10^−35^) and locations (*F*_2, 1008_ = 6.23, *p* = 0.002; *F*_3, 672_ = 18.47, *p* = 1.59×10^−11^). The 3-way interaction did not show any significance. The interaction between frequency bands and locations only showed the significance (*F*_12, 1008_ = 12.89, *p* = 6.89×10^−25^; *F*_18, 672_ = 4.01, *p* = 5.94×10^−8^) among the two-way interaction. We, therefore, investigated simple main effects of frequency bands by running separate two-way ANOVA for each level of the locations to infer which frequency band most greatly contributed to the prediction. Simple main effects of frequency bands for all levels of locations, except the fourth location, were significant (first location: *F*_6, 1239_ = 38.37, *p* = 1.34×10^−42^; *F*_6, 868_ = 12.81, *p* = 1.79×10^−13^; second location: *F*_6, 1239_ = 28.57, *p* = 1.03×10^−31^; *F*_6, 868_ = 20.82, *p* = 1.81×10^−22^; third location: *F*_6, 1239_ = 13.51, *p* = 1.84×10^−14^; *F*_6, 868_ = 11.56, *p* = 4.64×10^−12^). Multiple comparisons were conducted as shown in Figure 6. Multiple comparisons showed that the *δ* and *γ2* bands significantly contributed to the prediction more than any other frequency band in both monkeys. However, all frequency bands were needed for effective prediction because input dimensions were not reduced by the SLiR algorithm. No significant differences between *β1* and *β2* were observed for all levels of locations, showing that feature bands could be reduced by unifying *β1* and *β2.* The weight values of column #1 that were located in the most caudal part of the pre-central gyrus were slightly larger than those of the other columns located more rostral.

**Figure 7:**
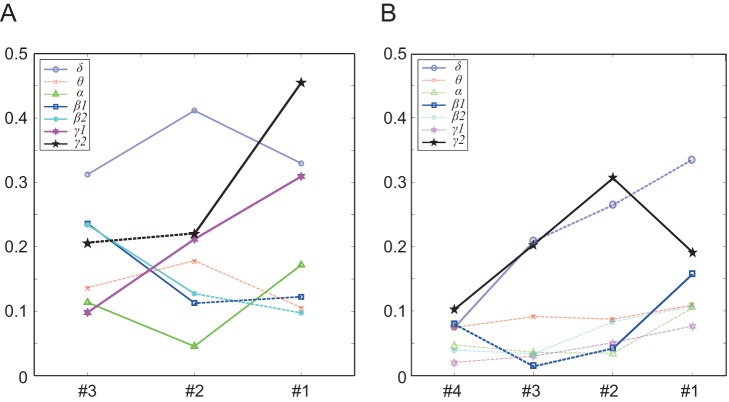
Simple main effect of electrode location contributing to EMG prediction. Each panel shows results of multiple comparisons among the locations for each frequency band level (A: monkey A, B: monkey B). Each marker displays the mean of the median weights. Faded lines show non-significant frequency bands. Solid lines represent a significant difference between weight values (*p*<0.001) and the dotted lines represent no significance. The # indicates the location of the ECoG electrodes.

We also conducted a two-way ANOVA to calculate simple main effects of the locations for each frequency band level. All simple main effects except *θ* level showed significance for monkey A (*δ*: *F*_2, 1239_ = 5.91, *p* = 0.003; *α*: *F*_2, 1239_ = 7.99, *p* = 3.5×10^−4^; *β1*: *F*_2, 1239_ = 9.11, *p* = 1.20×10^−4^; *β2*: *F*_2, 1239_ = 11.02, *p* = 1.78×10^−5^; *γ1*: *F*_2, 1239_ = 21.91, *p* = 4.30×10^−10^; *γ2*: *F*_2, 1239_ = 39.94, *p* = 1.46×10^−17^). The simple main effects of *δ*, *β1* and *γ2* showed significance for monkey B (*δ*: *F*_3, 868_ = 11.45, *p* = 3.18×10^−7^; *β1*: *F*_3, 868_ = 6.33, *p* = 3.99×10^−4^; *γ2*: *F*_3, 868_ = 20.08, *p* = 1.88×10^−12^). Results of multiple comparisons are summarized in Figure 7.

## Discussion

This study describes a novel attempt to predict multiple muscle activities from a small number of ECoG signals. This approach offers important insight regarding the presence of ample information in ECoG signals to predict time-varying muscle activities, whereas previous ECoG-based studies have tried to classify direction or intention of movement. The results clearly demonstrate that muscle activity time series and trial-to-trial variations of finger, hand, and arm muscles can be predicted from ECoG signals.

Previous studies using invasive methods have succeeded in the prediction of muscle activities through linear summation of the Ml firing rate [7], [28], [29], [42]–[45] or LFP [46]. Only one study, to our knowledge, has demonstrated that temporal activities of wrist muscles can be reconstructed from EEG cortical source currents, estimated from EEG sensor signals using a hierarchical Bayesian EEG inverse method [36]. Although their method could lead to drastic improvement in the non-invasive BMI area, it is still unknown whether it can be applied to sequential movements. In addition, their method has to solve an inverse problem, i.e., a projection from EEG sensors to current sources. A large number of current sources also increases the computational burden. In contrast, the present approach may be useful as a real-time force controller for BMI devices because it is fundamentally based on a simple filtering technique that addresses the z-score of frequency features of ECoG signals. This is the first report of a method for predicting multiple muscle activities from ECoG signals during natural forelimb movements.

Most EEG-based BMI studies have used one or two sensorimotor rhythms such as μ (8∼12 Hz) or *β* (14∼30 Hz) oscillations because the *γ* (>30 Hz) rhythm is often inconspicuous and neglected with a low pass filter, though Khan and Sepulveda [5] did use *γ* band EEG to discriminate four types of wrist motion. In ECoG-based BMIs, however, the *γ* rhythm has been widely used. In our studies, we identified the useful ECoG frequency bands associated with muscle activity. Analysis of the weight values for the frequency bands showed that contributions by the *δ*, *γ*, and *β* bands were significantly larger than those of the *θ* and *α* bands (e.g. Figure 6). This result corresponds to previous studies as well [21], [46]–[49].

Kinematic artifacts might have influenced the *δ* band to become the most contributing feature because the power spectrum of the movements had a peak in low frequency components (<2 Hz). These *δ* band features, however, are mainly derived from low frequency local field potentials (*lf*-LFPs) or movement event-related potentials (MRPs) in the frontal motor areas [4], [15], [50], [51]. The weights of the *δ* and *γ* bands were higher than those of the *θ* and *α* bands. This result coincides well with previous studies classifying of finger movement [52] and grasp type [22]. They reported that the accuracy of movement classification using power spectrums in high (75∼170 Hz) and low (<4 or 5 Hz) frequency bands was greater than that using intermediate frequency bands (6∼15 Hz and 17∼40 Hz).

It should be noted that no frequency band weights disappeared after applying the SLiR algorithm. This might indicate that all sensorimotor rhythms of ECoG encode muscle activity and are needed, at least at some degree, to predict them. Toro et al. (1994) reported that multi-joint arm movements were accompanied by a decrease in the spectral power of the 8∼12 Hz band in ECoG signals [53]. Previous works also reported a significant coherence between the M1 and EMG at the *β* band frequency [54], [55], [56]. Additionally, recent studies reported that higher frequency bands (>100 Hz [48]; 100∼200 Hz and 200∼400 Hz [51]) yielded better performance than the typically used *γ* band (30∼100 Hz). Therefore, we expect that the usage of all sensorimotor rhythms including the high frequency bands would contribute to the improved performance of an ECoG-based BMI.

In addition, the weight values of the electrodes located near the central sulcus were higher than those of the electrodes located more rostral. This result matches well with previous anatomical and physiological findings. CM cells that make monosynaptic connections to spinal motoneurons are located predominantly in anterior bank of the central sulcus [41]. The output from CM cells encodes muscle-activation patterns reflected in EMG activity [57]. Thus the frequency band features near the central sulcus may be the key to decoding muscle activities.

The primary advantage of the proposed method is that it can predict agonist and antagonist muscle activities during sequential movement tasks. If we can predict agonist and antagonist muscle activities, joint angle, torque, and stiffness can also be predicted using previously proposed methods [28], [29], [30]. This creates remarkable benefits, which would contribute to the realization of ECoG-based prosthetics.

## Supporting Information

Table S1 Summary of prediction accuracies for 10-fold cross validation of monkey A. Each cell except Avg. shows the CC or the nRMSE (mean ± STD) of 12 trials. Bold numbers indicate the best value in each test subset. The Avg. cells show the grand averages of mean and SEM. Bold numbers here indicate the best grand averages.
(DOCX)
