# Correction: Prediction of Muscle Activities from Electrocorticograms in Primary Motor Cortex of Primates

**DOI:** 10.1371/journal.pone.0092653

**Published:** 2014-03-27

**Authors:** The PLOS ONE Staff

In Figure 1B, the central sulcus line of subject B is incorrect. The authors have provided a corrected version of Figure 1 here.

**Figure 1:**
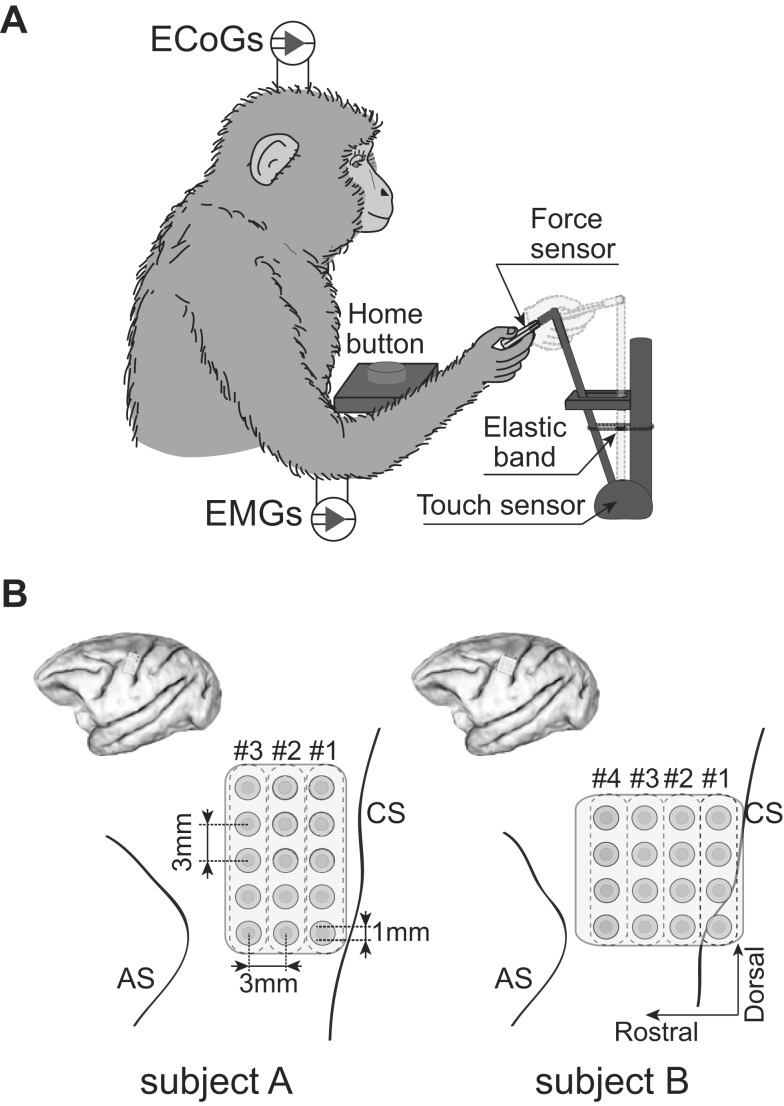
Behavioral task and ECoG electrode locations. A) Monkeys performed sequential right arm and hand movements, which consisted of reaching to a knob, grasping the knob with a lateral grip, pulling the knob closer, releasing the knob, and returning the hand to the home position, in a 3-D workspace. During the task, ECoG and EMG signals were recorded simultaneously. B) Schematic diagrams of ECoG electrode locations in left hemisphere. The planar-surface platinum electrode arrays were placed on the gyrus between the central sulcus (CS) and the arcuate sulcus (AS) in the primary motor area. The # indicates the location according to the column of ECoG electrodes.
