# Supplementary material for: Prediction of Muscle Activities from Electrocorticograms in Primary Motor Cortex of Primates
Source: PLoS One. 2012 Oct 24;7(10):e47992. doi: 10.1371/journal.pone.0047992 (PMC3480494; doi:10.1371/journal.pone.0047992)
Supplement: Table S1 — Summary of prediction accuracies for 10-fold cross validation of monkey A. Each cell except Avg. shows the CC or the nRMSE (mean ± STD) of 12 trials. Bold numbers indicate the best value in each test subset. The Avg. cells show the grand averages of mean and SEM. Bold numbers here indicate the best grand averages. (DOCX) [file pone.0047992.s001.docx]

Table S1. Summary of prediction accuracies for 10-fold cross validation of monkey A

|  | Test | TB | BB | EDC | ECU | APL | AP | FDP | FCU | FDS | PL | FCR | PT |
| --- | --- | --- | --- | --- | --- | --- | --- | --- | --- | --- | --- | --- | --- |
| CC | 1 | 0.50±0.15 | 0.59±0.14 | 0.60±0.07 | 0.41±0.19 | 0.60±0.13 | 0.48±0.13 | 0.54±0.17 | 0.47±0.18 | 0.48±0.18 | 0.45±0.15 | 0.66±0.12 | 0.50±0.21 |
|  | 2 | 0.47±0.19 | 0.67±0.08 | 0.65±0.13 | 0.44±0.25 | 0.63±0.18 | 0.52±0.20 | 0.58±0.13 | 0.55±0.14 | 0.49±0.20 | 0.50±0.21 | 0.60±0.09 | 0.52±0.14 |
|  | 3 | 0.55±0.11 | 0.62±0.10 | 0.68±0.12 | 0.50±0.21 | 0.62±0.08 | 0.57±0.15 | 0.63±0.11 | 0.61±0.16 | 0.63±0.10 | 0.54±0.10 | 0.63±0.15 | 0.57±0.12 |
|  | 4 | 0.59±0.12 | 0.64±0.09 | 0.63±0.11 | 0.50±0.16 | 0.66±0.09 | 0.57±0.13 | 0.58±0.15 | 0.55±0.16 | 0.60±0.13 | 0.56±0.15 | 0.61±0.12 | 0.35±0.22 |
|  | 5 | 0.52±0.14 | 0.62±0.12 | 0.69±0.08 | 0.44±0.20 | 0.60±0.14 | 0.50±0.18 | 0.56±0.15 | 0.51±0.16 | 0.54±0.17 | 0.43±0.23 | 0.52±0.23 | 0.38±0.18 |
|  | 6 | 0.57±0.15 | 0.66±0.08 | 0.66±0.13 | 0.43±0.18 | 0.58±0.16 | 0.48±0.17 | 0.55±0.12 | 0.52±0.12 | 0.58±0.17 | 0.49±0.15 | 0.64±0.10 | 0.46±0.17 |
|  | 7 | 0.59±0.12 | 0.64±0.09 | 0.63±0.11 | 0.50±0.16 | 0.66±0.09 | 0.57±0.13 | 0.58±0.15 | 0.55±0.16 | 0.60±0.13 | 0.56±0.15 | 0.61±0.12 | 0.35±0.22 |
|  | 8 | 0.58±0.13 | 0.66±0.14 | 0.69±0.08 | 0.55±0.13 | 0.61±0.13 | 0.51±0.15 | 0.58±0.16 | 0.56±0.12 | 0.56±0.13 | 0.57±0.08 | 0.65±0.13 | 0.53±0.17 |
|  | 9 | 0.59±0.09 | 0.65±0.08 | 0.66±0.11 | 0.50±0.20 | 0.64±0.08 | 0.57±0.14 | 0.60±0.13 | 0.57±0.13 | 0.60±0.12 | 0.59±0.10 | 0.66±0.14 | 0.51±0.18 |
|  | 10 | 0.58±0.13 | 0.67±0.10 | 0.73±0.10 | 0.55±0.13 | 0.69±0.09 | 0.58±0.17 | 0.64±0.09 | 0.58±0.14 | 0.56±0.18 | 0.54±0.13 | 0.67±0.07 | 0.54±0.13 |
|  | Avg. | 0.55±0.01 | 0.64±0.007 | 0.66±0.011 | 0.48±0.014 | 0.63±0.010 | 0.53±0.012 | 0.58±0.010 | 0.55±0.012 | 0.56±0.015 | 0.52±0.015 | 0.62±0.0.01 | 0.47±0.024 |
| nRMSE | 1 | 0.18±0.02 | 0.18±0.03 | 0.21±0.02 | 0.21±0.04 | 0.21±0.03 | 0.19±0.02 | 0.21±0.03 | 0.20±0.03 | 0.21±0.05 | 0.19±0.03 | 0.17±0.02 | 0.24±0.03 |
|  | 2 | 0.19±0.02 | 0.18±0.02 | 0.20±0.03 | 0.20±0.04 | 0.19±0.03 | 0.18±0.03 | 0.20±0.02 | 0.19±0.04 | 0.21±0.08 | 0.18±0.03 | 0.17±0.02 | 0.25±0.05 |
|  | 3 | 0.18±0.02 | 0.18±0.02 | 0.18±0.02 | 0.18±0.03 | 0.19±0.03 | 0.17±0.02 | 0.19±0.03 | 0.17±0.02 | 0.16±0.02 | 0.19±0.02 | 0.18±0.07 | 0.23±0.04 |
|  | 4 | 0.17±0.03 | 0.17±0.02 | 0.20±0.03 | 0.19±0.04 | 0.19±0.03 | 0.18±0.04 | 0.21±0.03 | 0.19±0.04 | 0.17±0.03 | 0.18±0.03 | 0.17±0.02 | 0.28±0.03 |
|  | 5 | 0.17±0.02 | 0.19±0.02 | 0.19±0.03 | 0.22±0.04 | 0.19±0.03 | 0.19±0.03 | 0.21±0.03 | 0.20±0.05 | 0.18±0.04 | 0.20±0.04 | 0.21±0.08 | 0.27±0.04 |
|  | 6 | 0.17±0.04 | 0.17±0.03 | 0.18±0.03 | 0.20±0.03 | 0.19±0.03 | 0.18±0.03 | 0.21±0.02 | 0.19±0.02 | 0.17±0.04 | 0.19±0.02 | 0.17±0.03 | 0.25±0.04 |
|  | 7 | 0.17±0.03 | 0.17±0.02 | 0.20±0.03 | 0.19±0.04 | 0.19±0.03 | 0.18±0.04 | 0.21±0.03 | 0.19±0.04 | 0.17±0.03 | 0.18±0.03 | 0.17±0.02 | 0.28±0.03 |
|  | 8 | 0.17±0.04 | 0.17±0.04 | 0.19±0.02 | 0.20±0.05 | 0.19±0.02 | 0.19±0.03 | 0.21±0.04 | 0.19±0.02 | 0.18±0.04 | 0.18±0.02 | 0.16±0.03 | 0.24±0.04 |
|  | 9 | 0.18±0.04 | 0.18±0.02 | 0.19±0.03 | 0.18±0.04 | 0.19±0.03 | 0.17±0.02 | 0.20±0.02 | 0.18±0.02 | 0.17±0.02 | 0.17±0.02 | 0.17±0.05 | 0.26±0.04 |
|  | 10 | 0.17±0.03 | 0.17±0.03 | 0.18±0.03 | 0.18±0.02 | 0.18±0.02 | 0.18±0.03 | 0.20±0.02 | 0.18±0.04 | 0.19±0.05 | 0.18±0.03 | 0.17±0.03 | 0.24±0.03 |
|  | Avg. | **0.17±0.002** | 0.18±0.001 | 0.19±0.003 | 0.19±0.004 | 0.19±0.002 | 0.18±0.002 | 0.20±0.002 | 0.19±0.003 | 0.18±0.005 | 0.18±0.003 | 0.18±0.004 | 0.25±0.005 |

Each cell except Avg. shows the CC or the nRMSE (mean ± STD) of 12 trials. Bold numbers indicate the best value in each test subset. The Avg. cells show the grand averages of mean and SEM. Bold numbers here indicate the best grand averages.
